# Publisher Correction: Medusozoan genomes inform the evolution of the jellyfish body plan

**DOI:** 10.1038/s41559-019-0905-3

**Published:** 2019-05-02

**Authors:** Konstantin Khalturin, Chuya Shinzato, Maria Khalturina, Mayuko Hamada, Manabu Fujie, Ryo Koyanagi, Miyuki Kanda, Hiroki Goto, Friederike Anton-Erxleben, Masaya Toyokawa, Sho Toshino, Noriyuki Satoh

**Affiliations:** 1 Marine Genomics Unit, Okinawa Institute of Science and Technology Graduate University, Onna, Okinawa Japan; 2 DNA Sequencing Section, Okinawa Institute of Science and Technology Graduate University, Onna, Okinawa Japan; 3 Zoological Institute, Christian-Albrechts-Universität, Kiel, Germany; 4 Seikai National Fisheries Research Institute, Nagasaki, Japan; 5 Tropical Biosphere Research Center, University of the Ryukyus, Sesoko, Okinawa Japan; 6 Present Address: Atmosphere and Ocean Research Institute, University of Tokyo, Kashiwa, Chiba Japan; 7 Present Address: Ushimado Marine Institute, Okayama University, Setouchi, Okayama Japan; 8 Present Address: Kuroshio Biological Research Foundation, Otsuki, Kochi Japan

**Keywords:** Genomics, Evolutionary developmental biology, Zoology

Correction to: *Nature Ecology & Evolution* 10.1038/s41559-019-0853-y, published online 15 April 2019.

The version of this article originally published was not open access, but should have been open access. The error has been corrected, and the paper is now open access with a CC-BY license.

